# Disrupted Lipid Metabolism in Multiple Sclerosis: A Role for Liver X Receptors?

**DOI:** 10.3389/fendo.2021.639757

**Published:** 2021-04-13

**Authors:** Inés Pineda-Torra, Sherrice Siddique, Kirsty E. Waddington, Rachel Farrell, Elizabeth C. Jury

**Affiliations:** ^1^ Centre for Cardiometabolic and Vascular Medicine, Department of Medicine, University College London, London, United Kingdom; ^2^ Centre for Rheumatology, Department of Medicine, University College London, London, United Kingdom; ^3^ Department of Neuroinflammation, Institute of Neurology and National Hospital of Neurology and Neurosurgery, University College London, London, United Kingdom

**Keywords:** liver X receptor, multiple sclerosis, lipid metabolism, nuclear receptor, cholesterol

## Abstract

Multiple sclerosis (MS) is a chronic neurological disease driven by autoimmune, inflammatory and neurodegenerative processes leading to neuronal demyelination and subsequent degeneration. Systemic lipid metabolism is disturbed in people with MS, and lipid metabolic pathways are crucial to the protective process of remyelination. The lipid-activated transcription factors liver X receptors (LXRs) are important integrators of lipid metabolism and immunity. Consequently, there is a strong interest in targeting these receptors in a number of metabolic and inflammatory diseases, including MS. We have reviewed the evidence for involvement of LXR-driven lipid metabolism in the dysfunction of peripheral and brain-resident immune cells in MS, focusing on human studies, both the relapsing remitting and progressive phases of the disease are discussed. Finally, we discuss the therapeutic potential of modulating the activity of these receptors with existing pharmacological agents and highlight important areas of future research.

## Introduction

Multiple sclerosis (MS) is a chronic degenerative disease of the central nervous system (CNS) and a major cause of neurological disability amongst young adults (1). The disease course is heterogeneous, characterized by acute onset neurological symptoms (relapses) and steady accrual of disability (progression). The underlying pathophysiology is complex and differences exist in the mechanisms causing relapse-predominant MS (RMS) and progressive neurodegeneration (either primary progressive where progression occurs from disease onset or secondary progressive where progression follows a period of relapsing disease) (2). In RMS, relapses are associated with auto-inflammatory processes driven by defects in immune regulation and activation and, migration of multiple effector immune cells across the blood brain barrier (BBB) into the CNS. Interactions between autoreactive immune cells and CNS resident cells, such as microglia and astrocytes, result in the release of inflammatory mediators that exacerbate localized inflammation. These inflammatory episodes resolve and lesions remyelinate, however subsequent neuronal degeneration can lead to persistent disability (3, 4).

The mechanisms driving accrual of disability in progressive MS are not well characterized but include neuro-axonal, oligodendrocyte and astrocyte damage leading to neurodegeneration. This is mediated by compartmentalized chronic inflammation within the CNS, involving the formation of CNS lymphoid-like structures and activation of CNS-resident innate cells (including microglia); notably, unlike RMS, the BBB is less permeable to immune cells migrating from the periphery (5–7).

Evidence supports a role for lipid metabolism (including changes in cholesterol, oxysterols, sphingolipids and fatty acids) not only in MS pathogenesis, but also as biomarkers of disease activity and progression and as treatment targets (8–14). One hypothesis is that abnormal lipid-mediated signaling in immune cells could contribute to MS pathogenesis (15). Lipid metabolism plays a crucial role in immune cell activation, differentiation and effector function (16). For example, activated T-cells have higher plasma membrane cholesterol (17) and fatty acid levels (18) and, fatty acid synthesis controls lineage differentiation into pro-inflammatory T-helper (Th)17 cells (19). Furthermore, modulation of plasma membrane lipid rafts, signaling microdomains in the plasma membrane enriched with lipids such as cholesterol and glycosphingolipids, influence immune cell differentiation and function (20, 21) with potentially pathogenic consequences (22). Conversely, manipulation of plasma membrane lipids can restore immune cell function in autoimmunity and cancer (23–25).

Interestingly, statins, inhibitors of the cholesterol biosynthesis enzyme 3-Hydroxy-3-Methylglutaryl-CoA Reductase-a widely used class of lipid lowering therapy, have been extensively studied in MS (26). Notably, a phase-II clinical trial showed that high dose simvastatin (CNS-penetrant statin) attenuated brain atrophy and disease progression without adverse effects in secondary progressive MS patients (27). A phase-III clinical trial is underway (MS-STAT2; NCT03387670, http://www.isrctn.com/ISRCTN82598726). Statins have pleiotropic effects on the immune system through the simultaneous promotion of Th2 differentiation, inhibition of Th1 mediated damage and reduction of neurotoxic pro-inflammatory molecules (28). Simvastatin also inhibits secretion of cytokines necessary for Th1 and Th17 differentiation in RMS patients (29) by inhibiting the interferon regulatory factor-4 transcription factor (30). Statins may also work through inhibition of mevalonate pathway-derived isoprenoids that mediate membrane association of certain signaling proteins, rather than direct inhibition of cholesterol itself (31, 32).

How disrupted lipid metabolism influences disease processes in MS remains uncertain. The lipid-activated nuclear receptors, liver X receptors (LXRs) and peroxisome proliferator-activated receptors (33, 34), are responsible for integration of lipid metabolism signaling in multiple immune and neuronal cell types, and could both play an important role (33, 35). This mini review presents evidence to support a role for LXRs in dysregulated lipid metabolism and immunopathogenesis in MS.

## Liver X Receptors

LXRs are nuclear transcription factors with key functions in lipid metabolism and cholesterol homeostasis (36–39). Two isoforms exist, LXRα and LXRβ, encoded by *NR1H3* and *NR1H2* genes respectively (40). They share 78% of their amino acid sequence identity but are differentially expressed; LXRα in metabolically active tissues (including liver, adipose tissue, macrophages, lung, intestine) while LXRβ is expressed ubiquitously. LXRs are activated by oxidized derivatives of cholesterol (oxysterols) (41–43) and intermediates of cholesterol biosynthesis (44, 45). Synthetic ligands for LXRs have been developed and used to understand LXR function, the most common being GW3965 and T0901317 (later reported to also act on other nuclear receptors) (46–49).

Cholesterol forms an essential component of cellular membranes and its oxysterol derivatives regulate many cellular processes. Cholesterol overload is toxic to cells, therefore pathways responsible for its generation are coupled to those responsible for cellular efflux (removal) and are tightly controlled, to ensure homeostasis (17). LXRs regulate intracellular lipid (including cholesterol) metabolism through a number of pathways including reverse cholesterol transport *via* the ATP binding cassette transporters (ABC)A1 (50) and ABCG1 (51) which promote cholesterol removal to the liver for catabolism and excretion by high density lipoprotein (HDL) particles. LXRs regulate the transcription of numerous genes involved in this process including, apolipoprotein-A1 (Apo-A1), apolipoprotein-E (Apo-E) (52, 53) and cholesteryl ester transfer protein (54). Other processes regulated by LXRs include; inducible degrader of the LDL receptor (55); Niemann Pick type-C proteins-1 and 2 involved in the lysosomal/late endosomal trafficking and recycling of intracellular lipids (56); fatty acid metabolism both *de novo* synthesis or through the Sterol Regulatory Element Binding Protein (SREBP)1, fatty acid synthase (FASN (57)) and fatty acid desaturation (FADS1, FADS2), elongation (elongation of very long-chain fatty acids protein) and phospholipid remodeling (Phospholipid transfer protein and lysophosphatidylcholine acyltransferase-3) (58–60).

**Figure 1:**
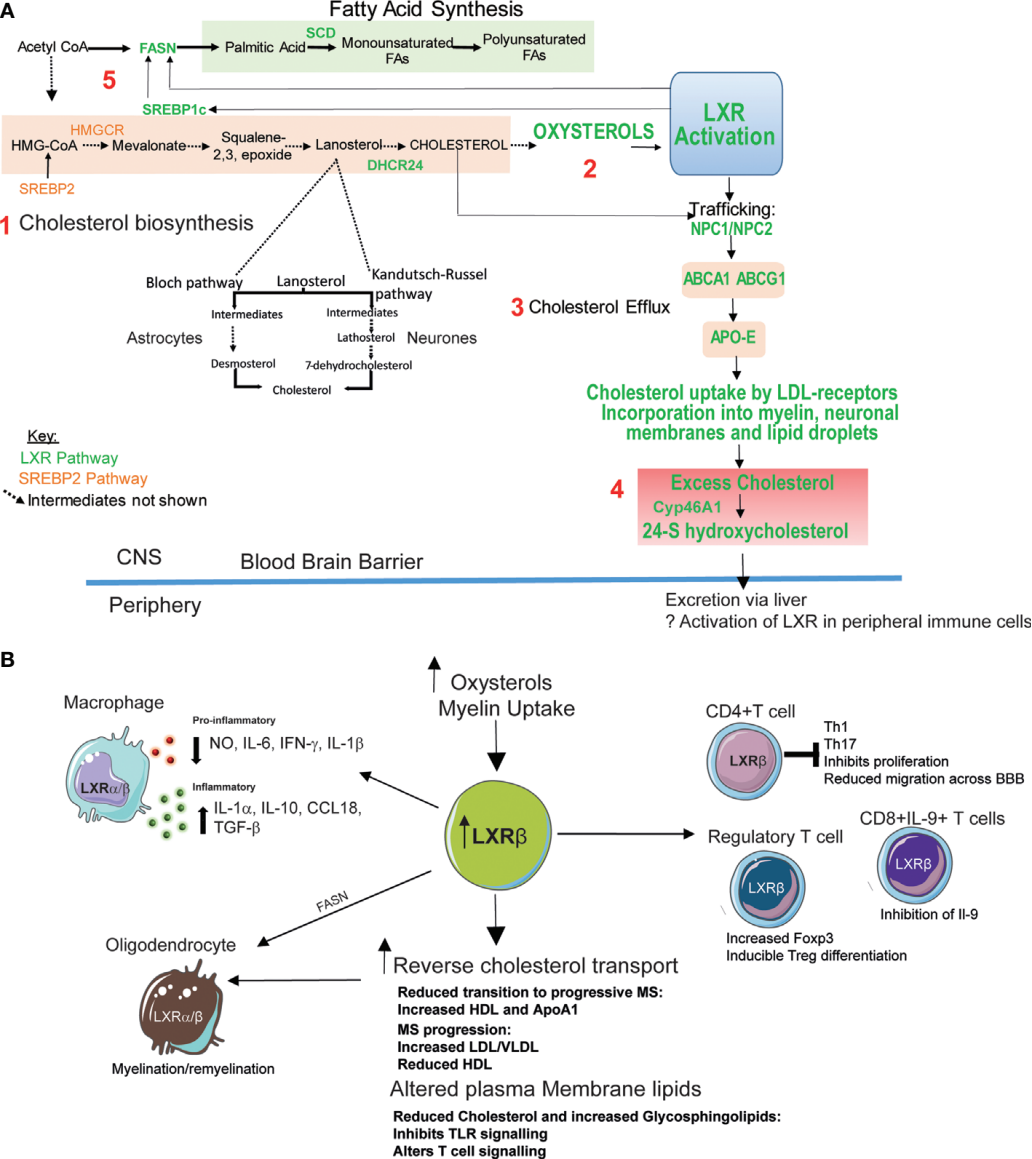
Potential therapeutic roles of LXR activation in MS. **(A)** Intracellular cholesterol levels in the brain are tightly regulated by two transcription factors (61): 1. Liver-X-receptor (LXR) and sterol response element binding-protein 2 (SREBP-2). SREBP2 upregulates genes involved in cholesterol biosynthesis. Cholesterol in the brain is produced *de novo* mainly by glial cells such as astrocytes using the Bloch pathway. Neurons which have a high cholesterol requirement produce less cholesterol *via* the Kandutsch-Russell pathway. 2. LXR is activated by by-products of cholesterol synthesis (oxysterols). 3. LXR activation promotes cholesterol export *via* intracellular cholesterol transporter Niemann Pick Type C1 and 2 (NPC1/NPC2), and ATP binding cassette (ABC) A1 and ABCG1 which efflux cholesterol from the plasma membrane to high density lipoprotein (HDL)-like lipoproteins including apolipoprotein-E (Apo-E). 4. Cholesterol is taken up by cells *via* lipoprotein receptors. Excess cellular cholesterol (potentially generated by neurodegeneration processes) is stored in lipid droplets or converted into oxysterols. 24-S hydroxycholesterol is the most abundant oxysterol in the brain and its production is catalyzed by the enzyme Cyp46A1 (cholesterol 24S-hydroxylase). 24-S hydroxycholesterol is able to cross the blood brain barrier to the periphery where it is degraded in the liver. 5. LXR also promotes fatty acid synthesis through its target genes SREBP1c, fatty acid synthase (FASN) and stearoyl-CoA desaturase (SCD). Plasma membrane levels of cholesterol and fatty acids can influence lipid rafts-membrane microdomains important for immune synapse formation and immune cell activation and function. Fatty acid (glycosphingolipid) abundance and composition can also influence plasma membrane fluidity (64). **(B)** LXRβ expression is elevated in peripheral blood mononuclear cells from MS patients potentially due to increased levels of oxysterols including 24S-hydroxycholesterol. Increased LXR activation can also be triggered by myelin uptake by glial cells in the central nervous system (CNS). LXR activation induces reverse cholesterol transport (**A**, 4). Patients with MS have altered lipoprotein profiles which may reflect defects in the efficacy of this process. MS progression is associated with reduced levels of high density lipoproteins (HDL)- responsible for effective cholesterol efflux. LXR activation also induces fatty acid and glycosphingolipid biosynthesis (**A**, 5). Changes in cellular cholesterol and glycosphingolipids can alter immune cell function by altering cell signaling and downstream functions including proliferation and cytokine production. In T-cells LXR activation reduces T-cell infiltration into the CNS (65) and inhibits naïve CD4+ T-cell differentiation towards an inflammatory Th17 phenotype (66) and suppressed IL-9 producing CD8+ T cells during anti-tumor responses (67). LXR activation is crucial for Treg function (68). LXR activation stimulates oligodendrocyte myelin production and remyelination processes (69). Mechanisms include stimulation of reverse cholesterol transport and fatty acid synthesis. LXR activation leads to the repression of inflammatory responses through the downregulation of pro-inflammatory genes including inducible nitric oxide synthase (NO), interleukin (IL)-1β, IL-6 and tumor necrosis factor-α. Myelin uptake by macrophages activates LXR and suppresses the production these pro-inflammatory mediators These myelin-laden macrophages, express high levels of anti-inflammatory IL-1-receptor-α, IL-10, CC-chemokine ligand-18 and transforming growth factor-β (70).

The brain contains 20% of body cholesterol and ~70-80% of cholesterol in the brain comprises an essential component of myelin in neuronal cells (61). The BBB prevents cholesterol transfer from the circulation into the brain, therefore brain cholesterol is synthesized *de novo* (62) *via* the 3-hydroxy-3-methylglutaryl-coenzyme-A reductase pathway. Cholesterol produced by glial cells is effluxed *via* ABCA1 to HDL-like molecules such as Apo-E, where it is taken up by LDL-receptors and other lipoprotein receptors in neurons (which have a high demand for cholesterol due to the large area of membrane in axons and dendrites). Intracellular cholesterol is transported *via* Niemann Pick type-C proteins. Conversely, excess cholesterol is eliminated *via* hydroxylation to 24(S)-hydroxycholesterol (catalyzed by cholesterol 24-hydroxylase), a polar oxysterol and the most abundant oxysterol in the brain, which crosses the BBB, enters the circulation, and is eliminated by the liver (61, 63) (**Figure 1A**).

Oligodendrocytes maintain myelination and remyelination processes within the CNS and LXR-regulated lipid metabolism pathways are crucial to their function (71, 72). CNS myelination is reduced in LXR knockout mice, conversely LXR activation stimulates oligodendrocyte maturation, myelin production and remyelination processes (69). Mechanisms include stimulation of reverse cholesterol transport *via* LXR target genes including ABCA1 and Apo-E, which restore remyelination in aged mice (12) and fatty acid synthesis; depletion of the LXR-target gene FASN blocked oligodendrocyte myelination and remyelination in the murine CNS (73).

### Cholesterol, Oxysterols and LXR in MS 

The relationship between disrupted serum cholesterol levels and adverse clinical outcomes in MS has been observed in several studies (74). Notably elevated apolipoprotein-B (Apo-B) (the major component of low/very low density lipoprotein cholesterol, LDL/VLDL) in clinically isolated syndrome (before confirmed MS diagnosis) correlated positively with increased Expanded Disability Status Scale (EDSS) indicating that cholesterol levels could serve as biomarkers for disease progression (74, 75), even accounting for age as a confounder. Similarly, in RMS, elevated serum LDL correlated positively with disease activity assessed by new MRI lesions (10, 11); increased LDL, total cholesterol and Apo-B levels were independently associated with higher EDSS score (9, 76); as were elevated VLDL subset levels (77). Conversely, high serum HDL was associated with reduced BBB injury and reduced inflammatory infiltrate in the cerebrospinal fluid (78). In RMS, increasing HDL and Apo-AI levels over time predicted a reduced likelihood of transition to secondary progressive disease and reduced brain atrophy (79). Also a greater reduction in HDL following interferon-β treatment in RMS patients predicted lower rates of future brain atrophy (10).

Differential patterns of oxysterol expression are also described in MS depending on the stage of disease (80, 81). Higher circulating oxysterols, notably, 24S-hydroxycholesterol, are thought to reflect elevated brain cholesterol metabolism and ongoing neurodegeneration (74, 81, 82). RMS patients progressing to secondary progressive disease over 5 years had higher CNS-derived serum 24S-hydroxycholesterol and Apo-B and reduced 7-ketocholesterol (83). While one study shows increased serum 7-ketocholesterol in patients with primary progressive disease (80). In older patients with RMS and those with primary progressive MS, serum 24S-hydroxycholesterol levels are low (84, 85) most likely due to increased brain atrophy and neuronal loss.

How changes in systemic cholesterol and oxysterols relate to LXR function in MS remain uncertain. Changes in oxysterol availability in MS (83) could lead to modulation of LXR signaling and influence subsequent immune cell function. For example, Th17 cells upregulate an enzyme that sulfates oxysterols (SULT2B1), thereby inactivating them as LXR ligands and driving preferential activation of RORγt (essential for Th17 function) instead of LXR (86). Also cholesterol/oxysterols are tightly suppressed in a subset of IL-9 producing CD8^+^ T cells to prevent transrepression of the *Il9* locus by LXR (67) and differentiated type-1 regulatory T-cells (Tregs) upregulate 25-hydroxycholesterol to limit IL-10 production (87).

LXRβ expression is elevated in peripheral blood mononuclear cells from MS patients compared to healthy controls supporting a role for LXR in immune cell dysregulation (88) and LXR signaling was upregulated in T-cells during the adoptive transfer EAE (experimental autoimmune encephalomyelitis) model of MS (89). Interestingly, absence of LXRα in brain endothelial cells in EAE resulted in more severe disease, increased BBB permeability and CNS inflammatory infiltrate (90).

MS patients are also characterized by other defects in lipid metabolism. A lipidomic analysis of CD4^+^ lymphocytes from MS patients identified altered phospholipids and elevated cardiolipins, potentially reflecting mitochondrial dysfunction (91). Glycosphingolipids (including ceramides and downstream metabolites hexosylceramide and lactosylceramide) are dysregulated in MS serum, plasma and immune cells (92–94). For example, decreased ceramides in white blood cells from MS patients were associated with impaired granulocyte-colony stimulating factor signaling and impaired neutrophil migration (93) and altered glycosphingolipid synthesis induced pathogenic inflammatory processes in astrocytes in a murine model of secondary progressive MS (95). Our recent work shows that LXR activation accelerates the conversion of ceramide to hexosylceramide (a key event in glycosphingolipid biosynthesis) in human CD4^+^T-cells. LXR stimulation regulated CD4^+^T-cell function in part by upregulating plasma membrane glycosphingolipids and reducing cholesterol thereby altering T-cell receptor-mediated signalling (96).

Collectively, these studies suggest that disrupted LXR function could be implicated in MS pathogenesis.

### Anti-Inflammatory Effects of LXRs in Immune Cells

LXR activation leads to the repression of inflammatory responses through the downregulation of pro-inflammatory genes including inducible nitric oxide synthase, interleukin (IL)-1β, IL-6 and tumor necrosis factor-α (97–100). This was thought to result from a transrepression mechanism involving SUMOylation of ligand-bound LXR. In macrophages, SUMOylation of LXR stabilizes corepressors on the nuclear factor kappa B (NF-κB) transcription factor, therefore dampening the transcription of target genes (101). However, a more recent study demonstrated LXRs ability to repress inflammatory genes in the absence of SUMOylation *via* the upregulation of the transmembrane cholesterol transporter ABCA1 which increases cholesterol efflux, alters plasma membrane lipid raft composition, and thereby inhibits Toll-like receptor signaling to downstream effectors NF-κB and mitogen-activated protein kinase (64).

The role of microglia (CNS-resident macrophages) in MS is complex; they can be both pathogenic (antigen presentation to T-cells and release of pro-inflammatory cytokines) and anti-inflammatory (clearing myelin debris and enabling remyelination) (102). LXR response genes ABCA1 and Apo-E are upregulated in microglia from active demyelinating MS lesions (103). The same study shows that myelin uptake induces production of 27-hydroxycholesterol oxysterol which activates LXRα and induces ABCA1 and Apo-E upregulation in human monocyte-derived macrophages. Myelin uptake by macrophages also activates LXRβ and suppresses the production the pro-inflammatory mediators nitric oxide and IL-6 and interferon-γ/IL-1β signalling (104). These myelin-laden macrophages, termed foamy macrophages, similar to lipid-laden macrophages present in atherosclerotic plaques, and derived from either resident microglia or infiltrating monocytes, have a distinct phenotype characterized by enhanced expression of genes involved in migration, phagocytosis and inflammation as well as genes involved in LXR signaling and cholesterol efflux. Moreover, murine foamy macrophages within MS lesions, defined by elevated HLA-DR and neutral lipid content, express high levels of anti-inflammatory IL-1-receptor-α, IL-10, CC-chemokine ligand-18 and transforming growth factor-β (70). Thus the anti-inflammatory effects of foamy macrophages arise from their response to phagocytosis of myelin, at least in part *via* LXR activation which suppresses pro-inflammatory mediator release and also inhibits T-lymphocyte proliferation (105).

LXR activation ameliorates EAE severity, potentially by reducing infiltration of T-cells into the CNS (65). Activation of LXRα and LXRβ can also inhibit naïve CD4^+^ T-cell differentiation towards an inflammatory Th17 phenotype. This occurs by activating SREBP1a and SREBP1c, which bind to the IL-17 promoter and the aryl hydrocarbon receptor (Ahr) (a positive regulator of Th17 differentiation), thus antagonizing Ahr-mediated IL-17 transcription (66). IL-17 suppression following LXR activation has been reproduced in splenocytes from the EAE model (106) and in in the context of other autoinflammatory diseases (107) such as Behcet’s disease. In murine models, LXR is crucial for Treg function by increasing Foxp3 expression and promoting inducible-Treg differentiation (68, 108).Together, these studies demonstrate that activation of LXR influences macrophage and T-cell differentiation and polarization (66, 104, 106, 107). These actions may be protective in the context of MS (**Figure 1B**).

### Therapeutic Activation of LXRs 

Due to their actions on lipid and cholesterol metabolism and the immune system, LXRs have attracted interest as therapeutic targets in neurodegenerative diseases (109, 110). Despite numerous studies showing the benefits of LXR agonism with the first generation of these compounds in experimental models, their translation to clinical practice has proven difficult. Systemic LXR activation promotes hepatic lipid accumulation (steatosis) and hypertriglyceridemia, both risk factors for cardiovascular disease, through the induction of *de novo* lipogenesis by LXRα in the liver (39). This prompted the development of a new generation of selective agonists, including selective LXRβ-agonists, tissue-selective agonists or agonists targeting the trans-repression/anti-inflammatory actions of LXRs (109) although, to our knowledge, none of these have been tested in preclinical models of MS (**Table 1**).

**Table 1:** Summary of synthetic LXR agonist effects.

Compound	Activity	Status	Disease/Model	Actions	Reference
**T0901317**	LXRα/β dual agonist	Preclinical	EAE (MS model)	Reduced CNS inflammation	(65, 66)
				Enhanced demyelination	
				Reduced Clinical severity	
		Preclinical	WT mice	Enhanced Myelin gene/protein expression	(69)
				Increased Oligodendrocyte maturation	
				Enhanced Remyelination	
**LXR-623**	LXRα/partial/β full agonist	Clinical Trial-Phase 1-Discontinued	Atherosclerosis	Adverse neurological effects	(111)
		Preclinical	Glioblastoma	Enhanced cell death	(112)
				Increased cholesterol depletion	
				Enhanced tumor regression	
				Increased Survival	
**BMS-852927**	LXRβ/selective partial agonist	Clinical Trial-Phase 1-Discontinued	Healthy subjects	Increased Cholesterol transport	(113)
				Enhanced Lipogenesis, triglycerides, LDL-C, apoB, apoE, CETP	
				Decreased circulating neutrophils	
**DMHCA/MePiPMHCA**	Transrepression-selective	Preclinical	Colitis, brain injury	Reduced inflammation	(114, 115)
				No induction of hepatic steatosis	
				SREBP1c inhibition	
**ATI-111**	Transrepression-selective	Preclinical	Atherosclerosis (Ldlr-null mice)	Reduced atherosclerosis	(116)
				Lowers plasma triglycerides and cholesterol	
				SREBP1c inhibition	

Macrophage-selective LXR agonists such as N,N-dimethyl-3β-hydroxycholenamide (DMHCA) and the desmosterol mimetic methylpiperidinyl-3β-hydroxycholenamide (MePipHCA) are examples of transrepression-dissociated agonists that avoid SREBP1c-driven hypertriglyceridemia (114, 115), as does the ATI-111 compound (116). By activating reverse cholesterol transport-related LXR target genes while blocking the processing of SREBP-1c, they act similarly to the endogenous ligands (e.g., desmosterol and oxysterols), which inhibit SREBP activation through actions in the endoplasmic reticulum (117). More recent reports on T0901317 and GW3965 showing LXR-independent non-genomic effects in pancreatic β cells by interfering with mitochondrial metabolism and cytosolic calcium concentrations (118) highlights the importance of testing the impact of novel LXR agonists in appropriate cellular or experimental systems lacking the receptors or alongside validated LXR antagonists. Whether this is replicated in other cellular systems will require further investigation (119).

Studies with the first generation of LXR agonists suggested their use as novel therapeutic agents for the treatment of MS. LXR activation in EAE dramatically ameliorates demyelination and inflammation in an LXR-dependent manner (65, 66). LXR activation in cerebellar cultures, using T0901317 and 25-hydroxycholesterol, enhanced expression of myelin-associated proteins, likely through transcriptional changes, while reverting the demyelinating phenotype in an LXR-dependent fashion (69). This study points to a potentially beneficial effect of LXR agonists on CNS remyelination and reduced neuronal damage. Notably, a loss of function mutation in the *NR1H3* gene encoding LXRα in patients presenting with a rare genetic form of severe progressive MS, indicates that aberrant LXR signaling could be involved in MS progression (120). The synthetic LXR agonist T0901317 restored LXR-mediated ABCA1 expression in a cell-line transfected with the mutant LXR, suggesting that pharmacological activation of LXRs could be beneficial in progressive MS.

Strategies for tissue specific delivery are important in addressing the challenge of delivering therapeutic agents across the BBB during progressive MS, when inflammation is largely restricted within the CNS. Interestingly, a highly brain penetrant partial LXRα/full LXRβ agonist (LXR-623) had beneficial effects in a murine model of glioblastoma (112). However, in healthy volunteers LXR-623 showed adverse neurological effects at higher doses (111). Another study in healthy subjects using LXRβ selective agonist BMS-852927, showed enhanced cholesterol transport in human macrophages but also SREBP1c-induced lipogenesis which had not been predicted from primate models (113). Thus limitations exist using animal models to predict therapeutic responses in humans. Differences in TLR4 regulation between human and rodent cells (121, 122), treatment duration in culture (121) and differing eicosanoid regulation by LXR (58, 123) have been reported and could underpin some limitations of the first generation LXR ligands.

Targeting LXRs in specific cell types or tissues could yield promising results for LXR-based therapeutics. For instance, atherosclerotic plaque-targeting nanoparticles encapsulating LXR ligands upregulate LXR target genes (including cholesterol efflux genes) and downregulate proinflammatory mediators in macrophages *in vitro* and reduce macrophage frequency and promote regression in plaques without adverse effects on hepatic lipid metabolism *in vivo* (124–128). Similar strategies show promise at inhibiting inflammation (129), promoting resolution of inflammation (130) and improving apoptotic cell clearance through phagocytosis using gold nanocages loaded with LXR ligands (131). These recent advances could expedite the path to clinical translation for LXR agonists. There is a lack of MS-focused research exploring beneficial effects of LXR activation using these novel delivery approaches but they have paved the way towards further exploration of LXR ligands as effective therapeutics against MS.

## Discussion

In conclusion, further investigation into the role of LXRs in MS immunopathogenesis is warranted. Activation of these receptors can modify the expression of cytokines and other immune mediators and polarize immune cells towards pro or anti-inflammatory phenotypes **(** **Figure 1**). In experimental models, LXR activation can ameliorate clinical symptoms. The role of LXRs has focused primarily on CD4^+^ T-cells and myeloid cells. However, the impact of lipid metabolism on other immune cells, particularly B-cells, is unexplored and could provide further insight into MS immunopathogenesis. Alternative strategies may focus on the modulation of immune cell function through lipid rafts.

Thus, dysregulated LXR-mediated pathways are likely to contribute to MS pathogenesis and provide a cohesive model describing the disease manifestations. A better understanding of LXRs in the context of MS is needed before their promising therapeutic potential can be fully realized.

## Author Contributions

SS researched and wrote a first draft of the review. KW, RF, IP-T, and EJ revised the manuscript. All authors contributed to the article and approved the submitted version.

## Funding

This work was supported by a project grant from the MS Society (Ref. 076) to IP-T, RF, and EJ.

## Conflict of Interest

The authors declare that the research was conducted in the absence of any commercial or financial relationships that could be construed as a potential conflict of interest.
